# Supplementary material for: Effects of Red and Blue Light on the Growth, Photosynthesis, and Subsequent Growth under Fluctuating Light of Cucumber Seedlings
Source: Plants (Basel). 2024 Jun 16;13(12):1668. doi: 10.3390/plants13121668 (PMC11207261; doi:10.3390/plants13121668)
Supplement: Supplementary file 1 [file plants-13-01668-s001.zip › plants-3019472-supplementary.pdf]

Table S1. Γ* and R_L under different treatments.

Treatments	Γ* (μmol·mol⁻¹)	R_L (μmol·m⁻²·s⁻¹)
**W**	41.66 ± 3.65 a	0.43 ± 0.10 c
**R**	35.75 ± 7.69 a	0.84 ± 0.12 a
**9R1B**	35.68 ± 5.08 a	0.32 ± 0.12 c
**7R3B**	33.05 ± 4.99 a	0.36 ± 0.17 c
**5R5B**	36.35 ± 4.20 a	0.76 ± 0.10 a
**3R7B**	39.81 ± 9.09 a	0.67 ± 0.07 ab
**1R9B**	31.03 ± 1.03 a	0.46 ± 0.13 bc
**B**	40.23 ± 9.23 a	0.84 ± 0.14 a

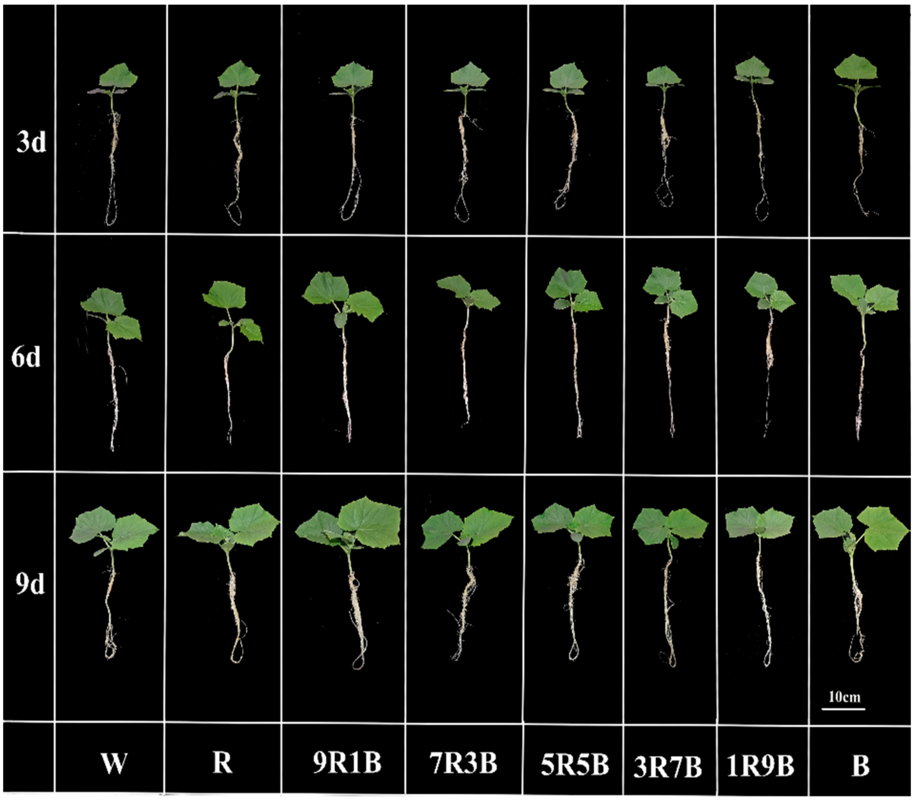

Figure S1. Effects of red and blue light on the morphological characteristics of cucumber seedlings.

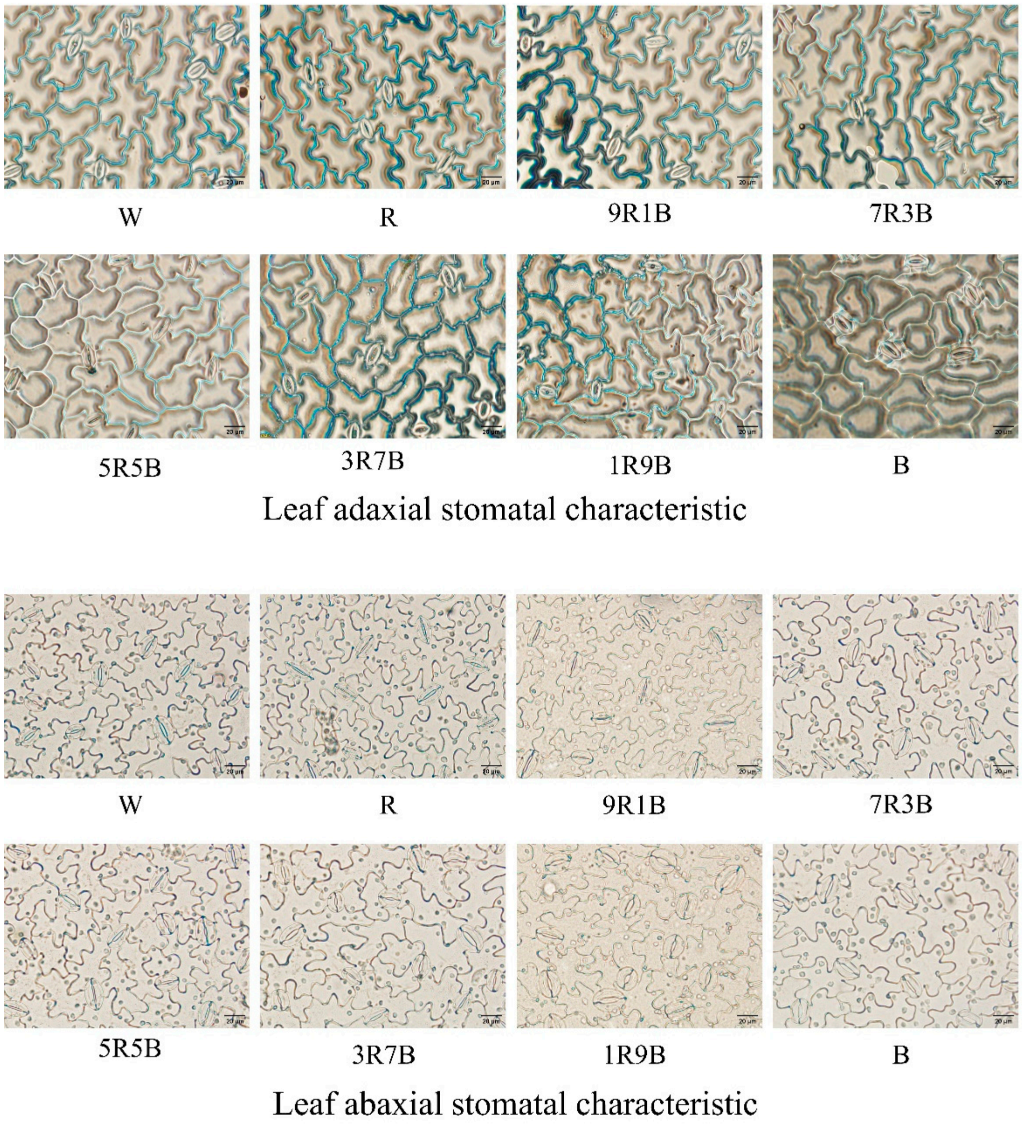

Figure S2. Stomatal characteristics of cucumber seedling leaves under different light treatment.

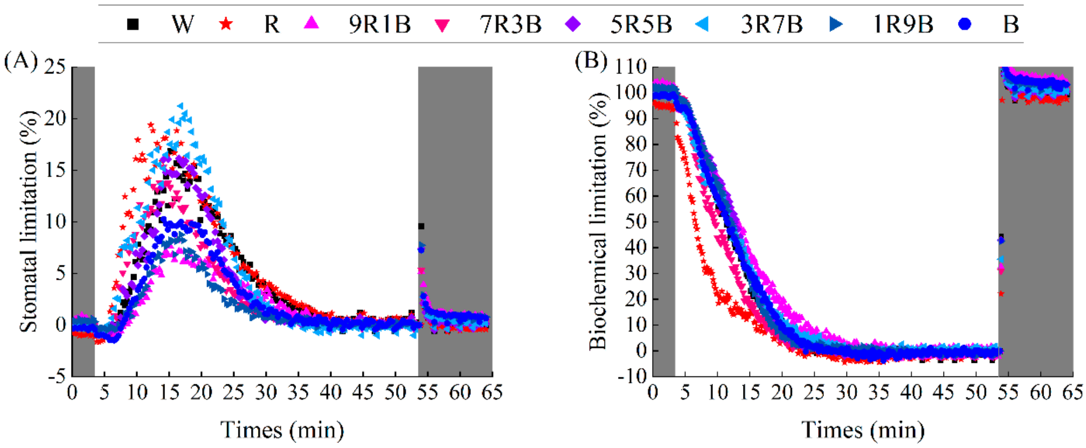

Figure S3. Limiting factors of photosynthesis during photoinduction.

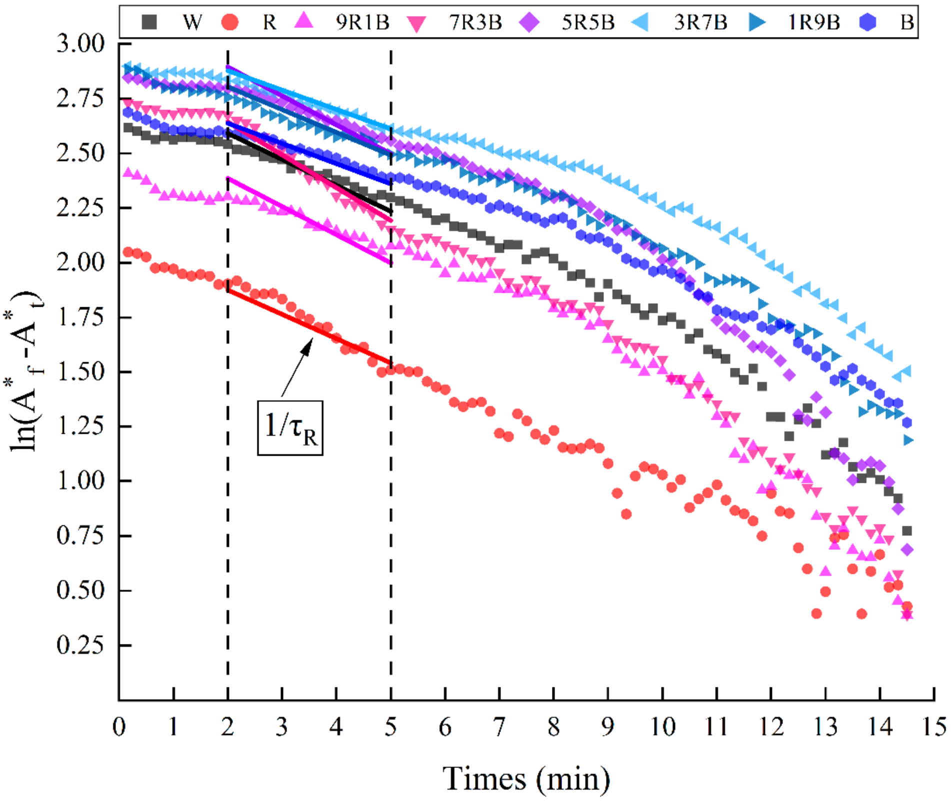

Figure S4. Calculation of Rubisco activation rate ($1/\tau_R$).

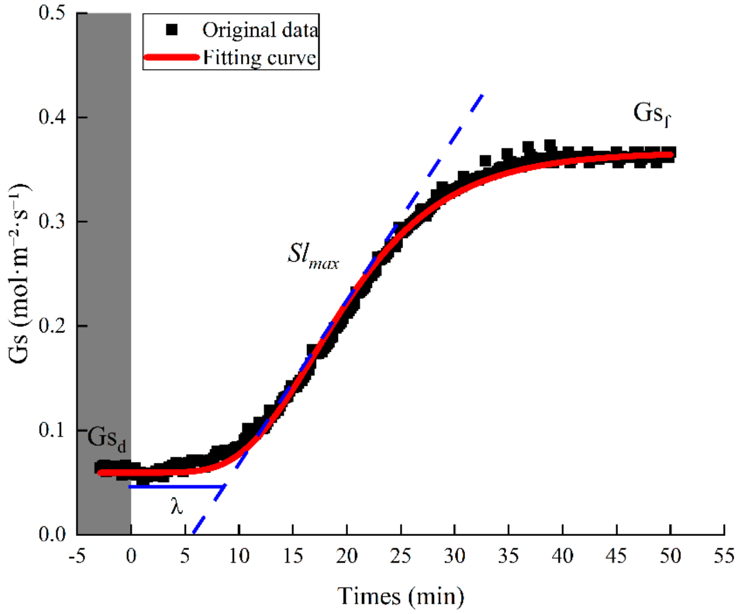

Figure S5. Calculation of the lag time of stomatal conductance in response to the increase of light intensity (λ) and the maximum increase rate of stomatal conductance (Sl_{max}). (Gs_d) was stomatal conductance under dark, Gs_f was stomatal conductance after light induction.

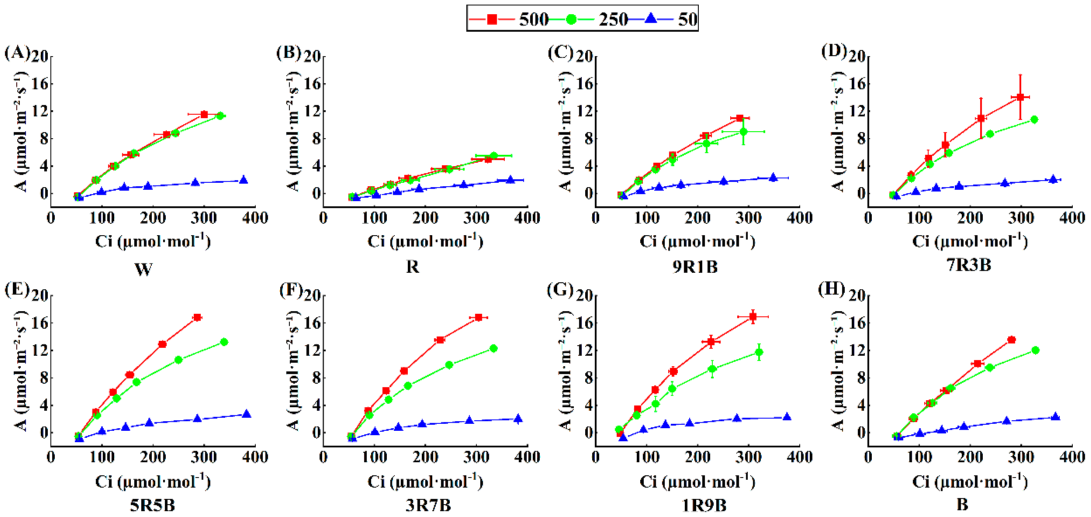

Figure S6. A-Ci curves under different light intensities.
